# Conditions of Birth and Early Childhood Developmental Risk for Mental Disorders

**DOI:** 10.1007/s10578-023-01549-2

**Published:** 2023-06-03

**Authors:** Felicity Harris, Kimberlie Dean, Oliver J. Watkeys, Kristin R. Laurens, Stacy Tzoumakis, Vaughan J. Carr, Melissa J. Green

**Affiliations:** 1 Discipline of Psychiatry and Mental Health, School of Clinical Medicine, University of New South Wales, Sydney, NSW Australia; 2 Justice Health & Forensic Mental Network, Matraville, NSW Australia; 3 Neuroscience Research Australia, Sydney, NSW Australia; 4 School of Psychology and Counselling, Queensland University of Technology, Brisbane, QLD Australia; 5 Griffith Criminology Institute, Griffith University, Southport, QLD Australia; 6 School of Criminology and Criminal Justice, Griffith University, Southport, QLD Australia; 7 Department of Psychiatry, Monash University, Melbourne, VIC Australia

**Keywords:** Perinatal risk factors, Familial risk factors, Developmental psychopathology, Child development, Data linkage

## Abstract

Distinct classes of children in the general population are at increased odds of later mental illness and other adverse outcomes according to patterns of early childhood developmental vulnerability. If certain risk factors known at the time of birth are reliably associated with membership in early childhood risk classes, then preventative interventions could be initiated in the earliest years of life. Associations between 14 factors known at the time of birth and membership in early childhood risk classes were examined in 66,464 children. Risk class membership was associated with maternal mental illness, parental criminal charges and being male; distinct patterns of association were shown for some conditions, for example, prenatal child protection notification was uniquely associated with misconduct risk’. These findings suggest that risk factors known at the time of birth could assist in very early detection of children who may benefit from early intervention in the first 2000 days.

## Introduction

The economic, health and social burden of mental illness is substantial [1], with approximately 45% of Australian adults likely to experience a mental illness in their lifetime [2], and 14% of young Australians (aged 4–17 years) estimated to have a mental illness within any 12-month period [3]. Substantive reduction of this burden could be achieved by the provision of adequate support services to those in greatest need, at the earliest opportunity [4–6]. Growing evidence indicates the perinatal period as a critical point of contact with the health system, in which the provision of targeted supports could prevent later mental health problems and associated adversity [7].

The importance of early intervention in the first 2000 days of life—the period from conception to age 5 years—is a key focus of strategic health policy frameworks globally, including in Australia [7]. These frameworks aim to facilitate access to appropriate supports and interagency services for vulnerable families during pregnancy and the early childhood period [8, 9]. However, doing so is dependent on service providers being able to identify which children (and families) are at greatest risk for mental health problems. This has been the focus of several lines of research, with evidence for associations between multiple risk indicators evident during the perinatal period, and both externalising and internalising symptomatology emerging at age ~ 5–6 years [10–15]. More recent research has established distinct clusters of familial and perinatal birth risk exposures that are associated with early child developmental vulnerabilities [16] as well as other adverse outcomes in later childhood (e.g., poor educational outcomes, child maltreatment, and police contact) [17, 18].

Distinct patterns of early childhood developmental vulnerability across multiple domains of function have been previously identified in a population cohort of 82,891 children who were assessed with the 2009 Australian Early Development Census (AEDC) in the state of New South Wales (NSW) [17]. The AEDC is a nationally administered triennial census of child development in the first year of full-time schooling (age ~ 5–6 years), completed by teachers who have undertaken a standardised training module. The AEDC assesses competency on five broad domains representing ‘social competence’, ‘emotional maturity’, ‘physical health and wellbeing’, ‘language and cognitive skills (school-based)’, and ‘communication skills and general knowledge’ that are underpinned by 16 subdomains. Using latent class analyses of early childhood developmental vulnerability (i.e., scores below the 10th percentile of the national distribution) on 16 subdomains of the AEDC, four risk classes were identified, including: a ‘pervasive risk’ class (4.2% of the population) characterised by a high probability of developmental vulnerability on all 16 AEDC subdomains; a ‘misconduct risk’ class (7.0%) characterised by high probability of developmental vulnerability on AEDC subdomains measuring hyperactive, inattentive and aggressive behaviour; a ‘mild generalised risk’ class (11.5%) characterised by low developmental vulnerability on most AEDC subdomains, but modest probabilities of developmental vulnerability on subdomains characteristic of academic achievement, engagement with learning, and communication, and; a ‘no risk’ class (77.0%) with virtually no developmental vulnerability on any AEDC subdomain [17, 19]. Membership in the ‘pervasive risk’ and ‘misconduct risk’ classes was associated with a threefold risk of mental disorder between the ages of 6–13 years, after accounting for socioeconomic disadvantage, notifications to child protection services, and parental mental illness [17]. All three risk classes have also been associated with an increased incidence of police contact up to age 13 years, most prominently for the ‘pervasive risk’ and ‘misconduct risk’ classes [18]. While the original LCA analyses also revealed moderate to strong associations between these risk classes and several demographic indices, perinatal events, and familial risk factors, these risk factors were not confined to information known at the time of birth.

The present study set out to examine a range of factors *known at the time of birth* in relation to membership in early childhood developmental risk classes. The overarching aim was to determine perinatal and familial characteristics known at birth that signify the need for indicated interventions (for families and/or infants) that could commence in the postnatal period before distinct patterns of developmental vulnerability are evident in offspring at school entry. Specifically, we estimated the associations between membership in the developmental ‘pervasive risk’, ‘misconduct risk’ or ‘mild generalised risk’ classes (relative to the ‘no risk’ class), and risk factors known at the time of birth, including demographic (child’s sex, socioeconomic status), perinatal (gestational age, low birthweight, maternal age, pregnancy complications, maternal smoking during pregnancy, number of previous pregnancies, antenatal visits) and other familial factors (parental history of mental illness or court charges, and prenatal child protection contact).

## Methods

### Cohort

**Fig. 1:**
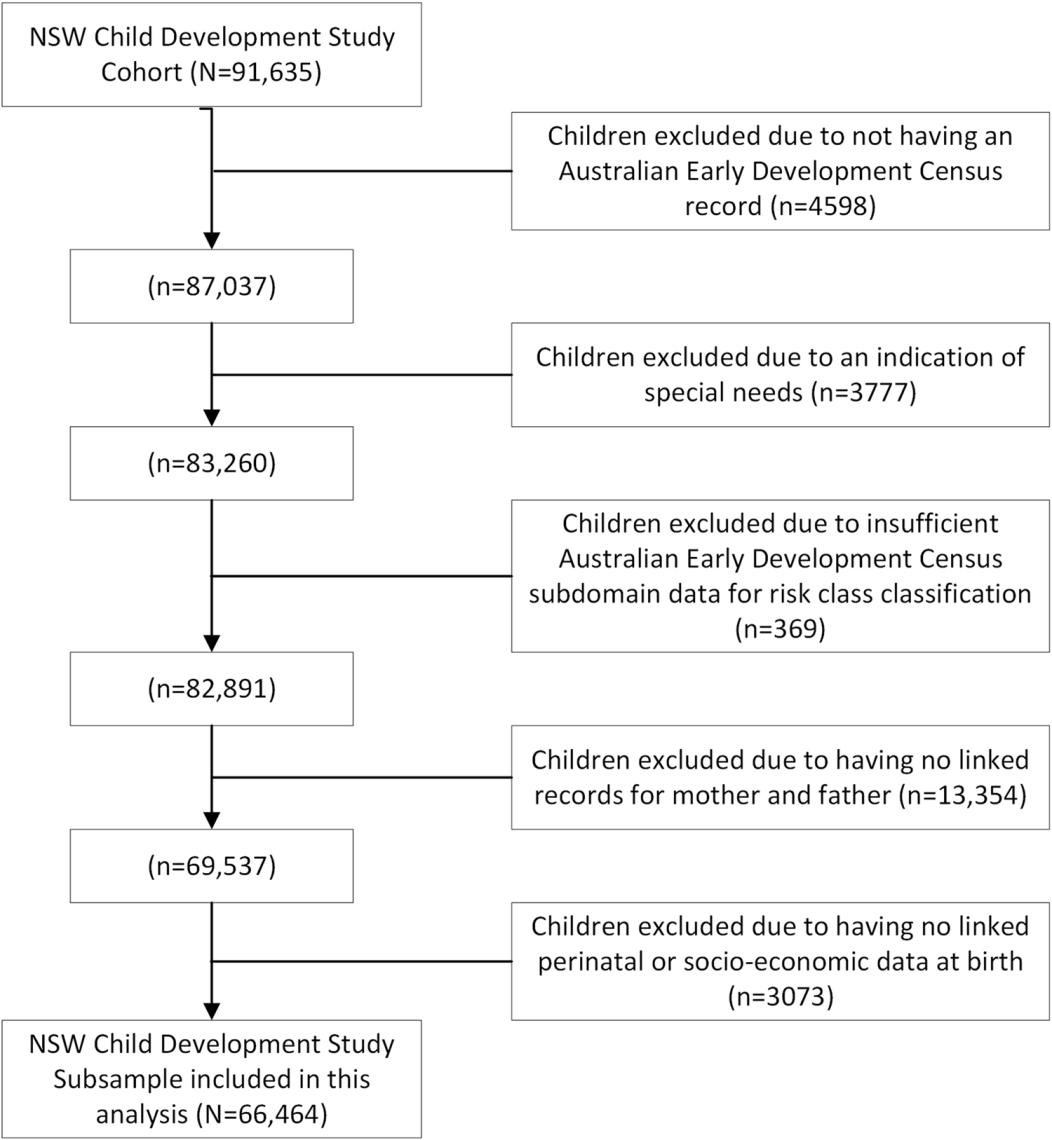
Sample selection flow chart

Participants were 66,464 children (50.8% boys) drawn from the NSW Child Development Study (NSW-CDS), with a mean age of 6.23 years (SD = 0.36) on 31 December 2009 (the year of AEDC administration) [20]. Inclusion criteria were valid AEDC subdomain data and complete data for all exposures, including the availability of both mother and father records (Fig. 1. Intergenerational data linkages were conducted by the Centre for Health Record Linkage (CHeReL; http://www.cherel.org.au), with optimal linkage rates (false-positive rates of approximately 0.5% for the child and parent cohorts). Parent records were available for children whose births were registered in NSW (i.e., 82.0% of the full NSW-CDS cohort of 91,635 children had mother records linked); further, 79.0% of the cohort had *both* mother and father records (since there is no requirement for a named ‘father’ on birth records). The sub-cohort of children included in this study is comparable with the entire NSW-CDS child cohort as well as with the general population on key demographics, including sex, age, socioeconomic disadvantage, geographical remoteness, Aboriginal and Torres Strait Islander background [20] The sample included 22.2% of children with a language background other than English (derived from relevant AEDC items), which is comparable to national statistics indicating that approximately 22% of the Australian population speak languages other than English in the home [21]. Ethical approval was granted by the NSW Population and Health Services Research Ethics Committee (PHSREC AU/1/1AFE112).

### Measures

#### Demographic Exposure Variables

*Male sex* (reference = female) was examined as a potential risk factor; each child’s sex was derived according to consensus across all available records. *Socioeconomic disadvantage* was measured according to quintiles of the Australian Bureau of Statistics’ Socioeconomic Index for Areas (SEIFA) Index for Relative Socio‐economic Disadvantage (IRSD) [22], derived from the mother’s residential postcode at birth (reference = least disadvantaged quintile).

#### Perinatal Exposure Variables

Perinatal events were recorded in the NSW Ministry of Health’s Perinatal Data Collection (PDC), the state-based surveillance system capturing perinatal and birth information. *Pregnancy complications* were represented as a single indicator of the presence of (any of) maternal diabetes mellitus, maternal hypertension, pre-eclampsia or gestational diabetes during pregnancy (reference = none). Maternal age included *young maternal age* (≤ 25 years) and *older maternal age* (> 36 years) as risk factors in reference to mothers aged 26–36 years. Antenatal visit included having *no antenatal visit* (none recorded) and having a *late first antenatal visit* (≥ 16 weeks gestation) in reference to an on-time first antenatal visit (< 16 weeks gestation) [23]. *Small for gestational age* was classified as a birthweight in the 10th percentile of Australian births relative to gestational age, in reference to a birthweight in the 11th–100th percentile [24]. *Preterm birth* was defined as a birth ≤ 37 weeks gestation (reference ≥ 38 weeks gestation) and having a record of any *maternal smoking during pregnancy* (reference = none) was selected as a potential risk factor. *Multiparity* (1–4 previous births) and *grand multiparity *(≥ 5 previous births) for previous pregnancies ≥ 20 weeks gestation were examined as risk factors (reference = no previous pregnancies).

#### Familial Exposure Variables

An indicator of *parental history of mental illness* (recorded in health data available prior to the child’s birth) was derived separately for mothers and fathers from primary or secondary diagnoses of mental disorder according to the International Statistical Classification of Diseases and Related Health Problems (10th edition, Australian Modification: ICD-10-AM). These diagnosis were recorded in the NSW Ministry of Health’s Admitted Patient Data Collection (covering all admitted patients’ services in NSW hospitals), Emergency Department Data Collection (emergency department presentations recorded in approximately 90 of 150 NSW public hospitals), and Mental Health Ambulatory Data Collection (recording mental health services provided to non-admitted patients).

Similarly, indicators of *parental history of court charges* prior to the child’s birth were derived separately for mothers and fathers from the NSW Bureau of Crime Statistics and Research Reoffending dataset (recording court appearances, including proven and unproven charges, for individuals who had been convicted of at least one prior criminal offence). *Prenatal child protection notification* (concerning risk to the child’s welfare known prior to birth) was derived from the NSW Department of Communities and Justice Child Protection Case Management System—Key Information Directory System (capturing children reported to the state child protection authority).

#### Developmental Risk (Age ~ 5–6 Years) Groups

Among the present study sample of 66,464 children, subdomains on the Australian Government Department of Education’s AEDC (2009) were used to classify 2475 (3.7%) in the ‘pervasive risk’ class, 4513 (6.8%) in the misconduct risk class, and 7146 (10.8%) in the ‘mild generalised risk’ class; a further 52,330, (78.7%) children were classified in the ‘no risk’ class, according to latent class analyses described previously [17]. The reliability and validity of the AEDC has been established [25, 26]; children who score below the 10th percentile of the national distribution in the 2009 census are regarded as developmentally vulnerable on that domain or subdomain [27].

### Statistical Analysis

A series of unadjusted and multivariable (adjusted for all exposures) multinominal logistic regression analyses were conducted using IBM SPSS Version 25.0 [28], to examine the associations between sociodemographic, perinatal, and familial exposure variables known at the time of birth, and the three early childhood risk classes (‘pervasive risk’, ‘misconduct risk’, and ‘mild generalised risk’), each compared to the ‘no risk’ class. The effect sizes for each covariate were reported as unadjusted (uOR) and adjusted odds ratios (aOR) and their 99% Confidence Intervals (99% CI), and regarded statistically significant when the 99% CI did not cross 1.00. The multicollinearity of exposures was acceptable (variable inflation factors ranged between 1.00 and 1.19).

## Results

### Sample Characteristics

The distribution of demographic, perinatal and familial risk indicators known at birth are presented in Table 1 for the whole cohort, and according to early childhood risk class membership. Between 46.8 and 74.6% of the children represented in each risk class were boys, with the ‘no risk’ class comprising the highest proportion of girls, and the ‘misconduct risk’ class comprising the highest proportion of boys. Socioeconomic disadvantage ranged from 17.0% among children with ‘no risk’ to 28.0% among children classified as ‘pervasive risk’. Of all risk classes, the group classified as ‘pervasive risk’ had the highest proportion of children exposed to perinatal factors, parental mental illness, and parental court charges. Overall, there was a low prevalence of prenatal child protection notification in all risk classes. Notably, 6.3% of the cohort were of Aboriginal or Torres Strait Islander background, among whom there was decreased representation in the ‘no risk’ class (5.1%) and increased representation in the three risk classes (i.e., 13.9% of ‘pervasive risk’, 9.3% of ‘misconduct risk’, and 10.3% of ‘mild generalised risk’ classes.

**Table 1:** Distribution of perinatal and parental birth exposures for the whole cohort, and stratified by early childhood risk class

Exposures known at the time of birth	Whole Cohort (n = 66,464)		Early childhood risk class								Chi-square (all p < .001)
			Pervasive risk (n = 2475)		Misconduct risk (n = 4513)		Mild generalised risk (n = 7146)		No risk (n = 52,330)		
	n	%	n	%	n	%	n	%	n	%	
Demographic factors											
Sex											Χ^2^(3) = 1870.24
Male	33,765	50.8	1729	69.9	3367	74.6	4161	58.2	24,508	46.8	
Female	32,699	49.2	746	30.1	1146	25.4	2985	41.8	27,822	53.2	
Socioeconomic disadvantage											Χ^2^(12) = 892.12
Quintile 1 (most disadvantaged)	12,378	18.6	692	28.0	929	20.6	1876	26.3	8881	17.0	
Quintile 2	12,925	19.4	582	23.5	941	20.9	1571	22.0	9831	18.8	
Quintile 3	14,239	21.4	537	21.7	969	21.5	1514	21.2	11,219	21.4	
Quintile 4	7297	11.0	227	9.2	468	10.4	694	9.7	5908	11.3	
Quintile 5 (least disadvantaged)	19,625	29.5	437	17.7	1206	26.7	1491	20.9	16,491	31.5	
Perinatal factors											
Maternal age											Χ^2^(6) = 799.38
≤25 years (young mother)	14,258	21.5	876	35.4	1296	28.7	2027	28.4	10,059	19.2	
26–35 years	42,122	63.4	373	15.1	641	14.2	990	13.9	8080	15.4	
≥ 36 years (older mother)	10,084	15.2	1226	49.5	2576	57.1	4129	57.8	34,191	65.3	
Pregnancy complications											Χ^2^(3) = 57.33
Pregnancy complications	7191	10.8	329	13.3	491	10.9	923	12.9	5448	10.4	
None	59,273	89.2	2146	86.7	4022	89.1	6223	87.1	46,882	89.6	
Maternal smoking during pregnancy											Χ^2^(3) = 989.86
Maternal smoking during pregnancy	8791	13.2	640	25.9	891	19.7	1426	20.0	5834	11.1	
None	57,673	86.8	1835	74.1	3622	80.3	5720	80.0	46,496	88.9	
Previous pregnancies											Χ^2^(6) = 535.07
Multiparity (1–4)	37,943	57.1	1479	59.8	2389	52.9	4432	62.0	29,643	56.6	
Grand multiparity (≥ 5)	861	1.3	103	4.2	62	1.4	212	3.0	484	0.9	
No previous pregnancies	27,660	41.6	893	36.1	2062	45.7	2502	35.0	22,203	42.4	
Size for gestational age											Χ^2^(3) = 162.44
Small for gestational age	7114	10.7	398	16.1	526	11.7	960	13.4	5230	10.0	
Not small for gestational age	59,350	89.3	2077	83.9	3987	88.3	6186	86.6	47,100	90.0	
Antenatal visit											Χ^2^(6) = 549.28
No antenatal visit	248	0.4	20	0.8	30	0.7	47	0.7	151	0.3	
Late (≥ 16 weeks) first antenatal visit	15,292	23.0	846	34.2	1122	24.9	2174	30.4	11,150	21.3	
Antenatal visit (< 16 weeks)	50,924	76.6	1609	65.0	3361	74.5	4925	68.9	41,029	78.4	
Gestational age											Χ^2^(3) = 133.90
Preterm birth	4034	6.1	239	9.7	295	6.5	582	8.1	2918	5.6	
Full-term birth	62,430	93.9	2236	90.3	4218	93.5	6564	91.9	49,412	94.4	
Familial factors											
Maternal mental illness											Χ^2^(3) = 319.05
Maternal history of mental illness	3120	4.7	231	9.3	347	7.7	461	6.5	2081	4.0	
No history	63,344	95.3	2244	90.7	4166	92.3	6685	93.5	50,249	96.0	
Paternal mental illness											Χ^2^(3) = 150.15
Paternal history of mental illness	1397	2.1	109	4.4	140	3.1	223	3.1	925	1.8	
No history	65,067	97.9	2366	95.6	4373	96.9	6923	96.9	51,405	98.2	
Maternal court charges											Χ^2^(3) = 609.12
Maternal history of court charges	3540	5.3	310	12.5	372	8.2	623	8.7	2235	4.3	
No history	62,924	94.7	2165	87.5	4141	91.8	6523	91.3	50,095	95.7	
Paternal court charges											Χ^2^(3) = 896.35
Paternal history of court charges	12,868	19.4	834	33.7	1233	27.3	1869	26.2	8932	17.1	
No history	53,596	80.6	1641	66.3	3280	72.7	5277	73.8	43,398	82.9	
Prenatal child protection notification											Χ^2^(3) = 78.78
Prenatal child protection notification	212	0.3	21	0.8	34	0.8	41	0.6	116	0.2	
None	66,252	99.7	2454	99.2	4479	99.2	7105	99.4	52,214	99.8	

### Unadjusted Models

The results of unadjusted multinomial logistic regression analyses are presented in Table 2. Among demographic risk factors, male sex showed the largest association with membership in the ‘misconduct risk’ class (uOR = 3.34, 99% CI 3.05, 3.65), with the ‘pervasive risk’ class showing the largest associations with socioeconomic disadvantage, followed by the ‘mild generalised risk’ class. While the largest unadjusted association was demonstrated between perinatal risk factors and the ‘pervasive risk’ class (specifically grand multiparity: uOR = 5.29, 99% CI 3.95,7.09), the familial risk factors showed consistently larger associations with ‘pervasive risk’ membership (see Table 2), although there was overlap among confidence intervals. Similar, yet smaller patterns of unadjusted association were evident in the ‘mild generalised’ risk class. In contrast the largest unadjusted associations for the ‘misconduct risk’ class were with the familial risk factors, with only some perinatal risk factors significantly associated.

**Table 2:** Unadjusted association between risk exposures at birth and early childhood risk class membership at age ~ 5–6 years

Exposures known at the time of birth	Early childhood risk class					
	Pervasive risk		Misconduct risk		Mild generalised risk	
	Wald	uOR (99% CI)	Wald	uOR (99% CI)	Wald	uOR (99% CI)
Demographic factors						
Sex						
Male	468.96	2.63 (2.35,2.95)	1164.20	3.34 (3.05,3.65)	323.07	1.58 (1.48,1.69)
Female		Ref		Ref		Ref
Socioeconomic disadvantage						
Quintile 1 (most disadvantaged)	297.77	2.94 (2.50,3.45)	61.63	1.43 (1.27,1.61)	522.97	2.34 (2.12,2.57)
Quintile 2	154.98	2.23 (1.89,2.64)	35.27	1.31 (1.16,1.47)	220.73	1.77 (1.60,1.95)
Quintile 3	81.30	1.81 (1.53,2.14)	13.77	1.18 (1.05,1.33)	108.32	1.49 (1.35,1.65)
Quintile 4	19.94	1.45 (1.17,1.80)	2.00	1.08 (0.94,1.25)	29.27	1.30 (1.15,1.47)
Quintile 5 (least disadvantaged)		Ref		Ref		Ref
Perinatal factors						
Maternal age						
≤ 25 years (young mother)	377.49	2.43 (2.16,2.73)	223.42	1.71 (1.56,1.88)	303.36	1.67 (1.55,1.80)
26–35 years		Ref		Ref		Ref
≥ 36 years (older mother)	17.49	1.29 (1.10,1.50)	1.27	1.05 (0.94,1.18)	0.15	1.01 (0.92,1.12)
Pregnancy complications						
Pregnancy complications	20.69	1.32 (1.13,1.54)	0.98	1.05 (0.92,1.19)	41.09	1.28 (1.16,1.41)
None		Ref		Ref		Ref
Maternal smoking during pregnancy						
Maternal smoking during pregnancy	454.34	2.78 (2.46,3.15)	284.81	1.96 (1.77,2.17)	440.95	1.99 (1.83,2.16)
None		Ref		Ref		Ref
Previous pregnancies						
Multiparity (2–4)	24.78	1.24 (1.11,1.39)	20.47	0.87 (0.80,0.94)	113.56	1.33 (1.24,1.42)
Grand multiparity (≥ 5)	214.51	5.29 (3.95,7.09)	5.52	1.38 (0.97,1.96)	255.01	3.89 (3.12,4.84)
No previous pregnancies		Ref		Ref		Ref
Size for gestational age						
Small for gestational age	92.85	1.73 (1.49,2.00)	12.57	1.19 (1.05,1.35)	79.15	1.40 (1.27,1.54)
Not small for gestational age		Ref		Ref		1.00 (Reference)
Antenatal visit						
No antenatal visit	25.87	3.38 (1.82,6.26)	19.49	2.43 (1.45,4.07)	32.28	2.59 (1.68,3.99)
Late (≥ 16 weeks) first antenatal visit	227.15	1.93 (1.73,2.17)	32.48	1.23 (1.12,1.35)	302.81	1.62 (1.51,1.75)
Antenatal visit (< 16 weeks)		Ref		Ref		Ref
Gestational age						
Preterm birth	70.49	1.81 (1.51,2.17)	7.17	1.18 (1.01,1.39)	73.95	1.50 (1.33,1.70)
Full-term birth		Ref		Ref		Ref
Familial factors						
Maternal mental illness						
Maternal history of mental illness	157.17	2.49 (2.06,3.00)	134.79	2.01 (1.72,2.35)	92.23	1.67 (1.45,1.91)
No history		Ref		Ref		Ref
Paternal mental illness						
Paternal history of mental illness	82.61	2.56 (1.96,3.34)	39.18	1.78 (1.40,2.26)	59.18	1.79 (1.47,2.18)
No history		Ref		Ref		Ref
Maternal court charges						
Maternal history of court charges	327.25	3.21 (2.72,3.79)	144.19	2.01 (1.73,2.34)	260.28	2.14 (1.90,2.42)
No history		Ref		Ref		Ref
Paternal court charges						
Paternal history of court charges	420.45	2.47 (2.20,2.77)	290.08	1.83 (1.67,2.00)	342.80	1.72 (1.60,1.86)
No history		Ref		Ref		Ref
Prenatal child protection notification						
Prenatal child protection notification	32.09	3.85 (2.09,7.11)	39.45	3.42 (2.06,5.66)	27.47	2.60 (1.62,4.15)
None		Ref		Ref		Ref

### Adjusted Model

**Fig. 2:**
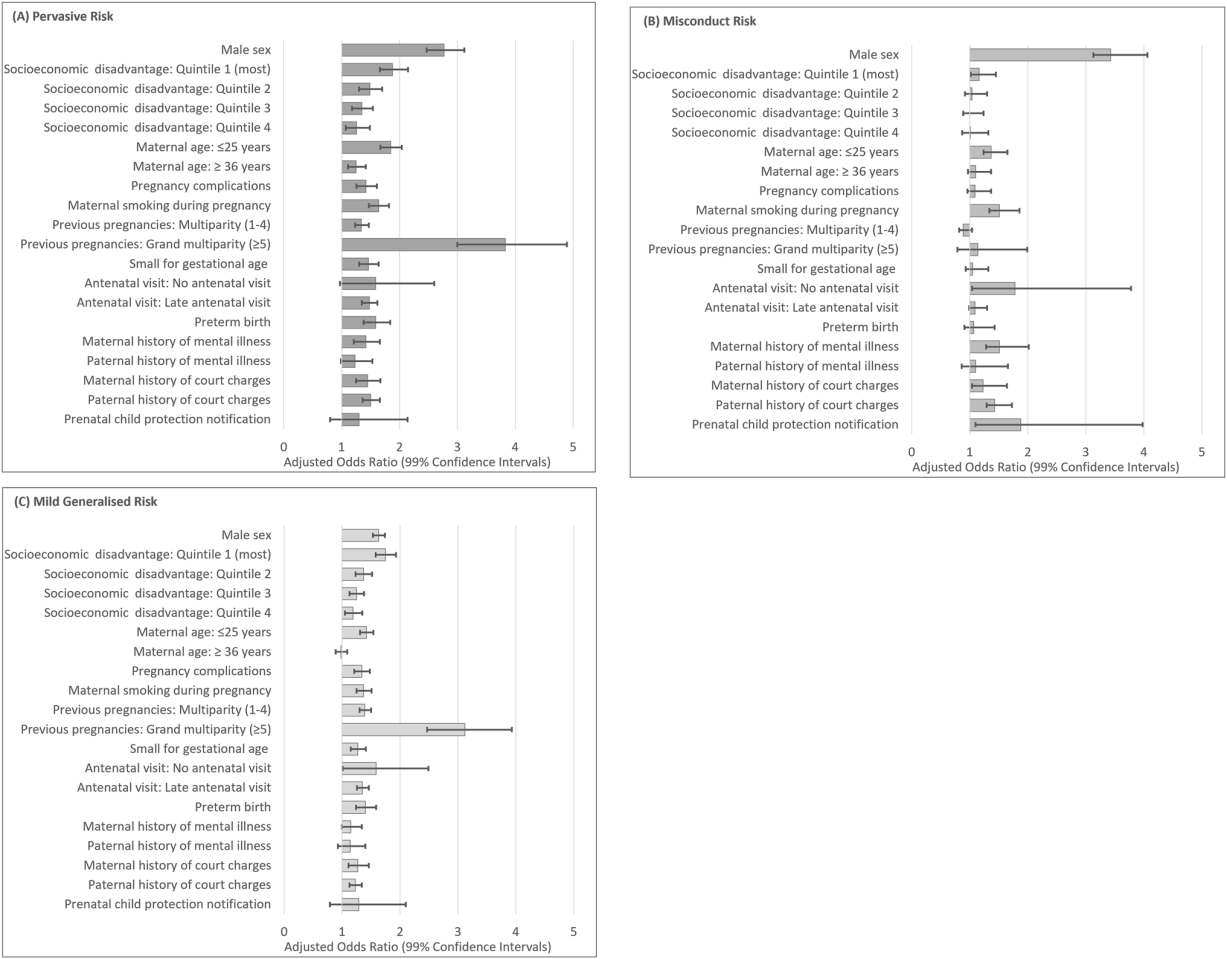
Adjusted association between early childhood risk class membership at age ~ 5–6 years and risk exposures at birth; bars indicate adjusted odds ratios and error base indicate 99% confidence intervals

The results from the adjusted multinominal logistic regression analysis are presented in Table 3, and graphically in Fig. 2. The magnitude of all associations except *male sex* decreased in the adjusted model, and *socioeconomic disadvantage* retained significant associations with each of the early childhood risk classes. When examining the pattern of associations within each risk-class in the multivariable model, the ‘misconduct risk’ class alone maintained a significant association with *prenatal child protection notification* (aOR = 1.88, 99% CI 1.10, 3.20)*,* as the largest effect for a familial risk factor at birth; this was followed by smaller effects for *young maternal age*, *maternal smoking during pregnancy*, *grand multiparity, no antenatal visit, maternal mental illness*, and *maternal and paternal history of court charge*s (see Table 3). In contrast, membership in the ‘pervasive risk’ class had the strongest associations among the perinatal risk factors (ranging from aOR = 1.25, 99%: CI 1.07, 1.47 for *older mother* to aOR = 3.83, 99%: CI 2.78, 5.27 for *grand multiparity*). A similar pattern of associations with the ‘mild generalised risk’ class where perinatal risk factors (significant associations ranging from aOR = 1.27, 99% CI 1.15,1.41 for *small for gestational age* to aOR = 3.12, 99% CI 2.47, 2.93 for *grand multiparity*) generally had larger effects than the familial risk factors. These findings must be interpreted in the context of some overlap between confidence intervals for each risk factor in the adjusted model.

**Table 3:** Adjusted association between risk exposures at birth and early childhood risk class membership at age ~ 5–6 years

Exposures known at the time of birth	Early childhood risk class					
	Pervasive risk		Misconduct risk		Mild generalised risk	
	Wald	aOR (99% CI)	Wald	aOR (99% CI)	Wald	aOR (99% CI)
Demographic factors						
Sex						
Male	507.40	2.77 (2.47,3.12)	1205.23	3.43 (3.13,3.76)	356.07	1.63 (1.53,1.74)
Female		Ref		Ref		Ref
Socioeconomic disadvantage						
Quintile 1 (most disadvantaged)	91.56	1.88 (1.59,2.24)	9.28	1.16 (1.02,1.31)	203.59	1.75 (1.58,1.93)
Quintile 2	34.08	1.49 (1.25,1.77)	0.82	1.04 (0.92,1.18)	60.97	1.37 (1.23,1.52)
Quintile 3	19.67	1.35 (1.13,1.61)	0.01	1.00 (0.89,1.13)	31.18	1.25 (1.13,1.38)
Quintile 4	7.60	1.26 (1.02,1.57)	0.05	1.01 (0.87,1.18)	12.51	1.19 (1.05,1.35)
Quintile 5 (least disadvantaged)		Ref		Ref		Ref
Perinatal factors						
Maternal age						
≤ 25 years (young mother)	146.55	1.85 (1.62,2.11)	62.71	1.37 (1.24,1.52)	117.29	1.42 (1.31,1.54)
26–35 years		Ref		Ref		Ref
≥36 years (older mother)	12.96	1.25 (1.07,1.47)	3.84	1.10 (0.97,1.24)	0.20	0.98 (0.89,1.09)
Pregnancy complications						
Pregnancy complications	31.65	1.42 (1.21,1.67)	2.87	1.09 (0.96,1.24)	56.30	1.34 (1.21,1.48)
None		Ref		Ref		Ref
Maternal smoking during pregnancy						
Maternal smoking during pregnancy	66.64	1.57 (1.36,1.81)	82.64	1.51 (1.34,1.69)	74.12	1.37 (1.25,1.51)
None		Ref		Ref		Ref
Previous pregnancies						
Multiparity (1–4)	40.39	1.34 (1.19,1.51)	12.58	0.89 (0.82,0.97)	139.20	1.39 (1.30,1.50)
Grand multiparity (≥ 5)	116.83	3.83 (2.78,5.27)	0.84	1.14 (0.79,1.64)	159.36	3.12 (2.47,3.93)
No previous pregnancies		Ref		Ref		Ref
Size for gestational age						
Small for gestational age	40.72	1.46 (1.25,1.70)	1.05	1.05 (0.93,1.20)	38.63	1.27 (1.15,1.41)
Not small for gestational age		Ref		Ref		Ref
Antenatal visit						
No antenatal visit	3.34	1.59 (0.83,3.03)	7.69	1.78 (1.04,3.04)	6.98	1.59 (1.01,2.49)
Late (≥ 16 weeks) first antenatal visit	73.81	1.48 (1.32,1.67)	4.98	1.09 (0.99,1.20)	110.07	1.35 (1.26,1.46)
Antenatal visit (< 16 weeks)		Ref		Ref		Ref
Gestational age						
Preterm birth	39.99	1.59 (1.32,1.92)	1.19	1.07 (0.91,1.27)	48.19	1.40 (1.24,1.59)
Full-term birth		Ref		Ref		Ref
Familial factors						
Maternal mental illness						
Maternal history of mental illness	19.24	1.42 (1.15,1.74)	40.71	1.51 (1.28,1.79)	6.41	1.15 (1.00,1.34)
No history		Ref		Ref		Ref
Paternal mental illness						
Paternal history of mental illness	3.57	1.23 (0.93,1.64)	1.04	1.10 (0.86,1.42)	2.70	1.14 (0.93,1.40)
No history		Ref		Ref		Ref
Maternal court charges						
Maternal history of court charges	25.22	1.45 (1.20,1.75)	9.76	1.23 (1.04,1.45)	21.15	1.27 (1.11,1.46)
No history		Ref		Ref		Ref
Paternal court charges						
Paternal history of court charges	64.77	1.50 (1.32,1.71)	80.85	1.43 (1.29,1.59)	40.25	1.23 (1.13,1.34)
No history		Ref		Ref		Ref
Prenatal child protection notification						
Prenatal child protection notification	1.10	1.30 (0.68,2.50)	9.26	1.88 (1.10,3.20)	1.79	1.29 (0.79,2.10)
None		Ref		Ref		Ref

## Discussion

In this large population study, two patterns of risk factors evident at the time of the child’s birth were associated with membership in distinct developmental risk classes at age ~ 5–6 years. First, exposure to several perinatal factors (e.g., *grand multiparity*, *young maternal age, maternal smoking during pregnancy*) was strongly associated with membership in all risk classes, with *male sex* as one of the strongest risk factors for membership in all risk classes. However, the pattern of associations observed for membership of the ‘misconduct risk’ class was somewhat different to the other risk classes, with exposures to familial factors known at the time of birth (e.g., *parental mental illness, parental court charges* and *prenatal child protection notifications*) having a generally greater magnitude of association with this class than perinatal factors. These findings could help to inform the targeted provision of preventative interventions in the earliest stages of life, to avert poor outcomes for children at risk of mental disorders [17], and other social adversities such as contact with the justice system [18]. In particular, these findings contribute to the body of research which informs the allocation of services to vulnerable families in the first 2000 days of their child’s development, before patterns of early developmental vulnerabilities are observable.

The distinct pattern of familial factors associated with the ‘misconduct risk’ class included *maternal history of mental illness, maternal* and *paternal history of court charges*, with small or no effects for perinatal factors (e.g., *multiparity and grand multiparity, small for gestational age, no antenatal visit, preterm birth*), and *prenatal child protection exposure* only maintaining association with ‘misconduct risk’ when all other factors were considered. This suggests that the behavioural profile of these children, who might carry a less adverse neurodevelopmental load than the other two classes (as suggested by minimal developmental vulnerabilities in areas characteristic of academic learning and motor function) may be more strongly determined by adverse social environments. The second pattern of risk factors associated with both the ‘pervasive risk’ and, to a lesser extent, ‘mild generalised risk’ classes was comprised of a combination of familial, perinatal and socioeconomic birth-risk factors; membership in these classes showed strong associations with perinatal indices (e.g., *grand multiparity, younger mother* and *maternal smoking during pregnancy*) and small or moderate associations with familial (e.g., *maternal history of mental illness, maternal and paternal history of court charges*) and socioeconomic (e.g., *socioeconomic disadvantage*) indices. These two patterns of risk may be detectable among expectant mothers receiving prenatal care and may thus assist to inform the earliest provision of targeted supports according to specific needs.

The NSW child protection authority delivers specific programs for vulnerable families involved with child protection services in the prenatal period [29], and Australia’s state-based health services administer voluntary universal health home visiting programs to provide support within the first two weeks of childbirth; home visits by nurses identify additional support needs and facilitate engagement with early intervention services during the postnatal period [30]. Expansion of these specialist services to identify families presenting with the factors associated with the ‘misconduct risk’ profile in childhood could potentially avert poor behavioural outcomes for the child early in development by enabling the family access to additional parenting support and suitable health services. Similarly, better access to early childhood education services for all children may be particularly beneficial to those who later show ‘pervasive risk’ and ‘mild generalised risk’ profiles of development at school entry, especially if they are also accompanied by enhanced parenting support and easy access to specialist health services. Early childhood education services are known to mitigate against poor developmental outcomes [31], including academic underachievement (which form part of the ‘pervasive risk;’ and ‘mild generalised risk’ profiles) in children experiencing adversity [32]. Indeed, a recent Australian study suggests that children with higher cumulative exposure to perinatal and sociodemographic risk factors early in life, similar to those associated with ‘pervasive risk’ and ‘mild generalised risk’ profiles, have a pattern of lower use, in early childhood education and health services [33]. Facilitating access to these programs for all vulnerable families should be a focus of health and social policy in Australia.

The current findings depart from previously reported associations between these early childhood risk classes and a range of risk factors (e.g., child protection services contact, small for gestational age, and parental mental illness) occurring before age ~ 5–6 year﻿s [19] which were associated with all risk classes to a similar degree. In the present study, with risk factors restricted to perinatal events and familial indicators *known at the time of birth*, we observed an emergence of two patterns of risk factors associated with either the ‘misconduct risk’ group or the ‘pervasive risk’ and ‘mild generalised risk’ groups. Moreover, the present study included additional perinatal factors that were not investigated in the earlier study, examined the effects of maternal and paternal risk factors separately, and used a more comprehensive index of child protection contacts (the earlier study was restricted to substantiated child protection reports, including out-of-home-care placements). However, the notable difference in findings for child protection contacts (which showed the strongest association for all three risk classes after male sex in the earlier study, but was associated only with the ‘misconduct risk’ class in the current study), are likely due to the lower portion of children (i.e., 0.3%) here with a prenatal child protection report known at birth compared to those (3.1%) who had been the subject of substantiated reports by age 5 years [19]. These less common prenatal child protection reports may reflect complex issues related to substance use, domestic violence, transgenerational trauma and mental ill-health, affecting the capacity of primary caregivers to adequately care for the child [29]. Finally, while previous studies have reported small to moderate associations between parental mental illness and early developmental vulnerabilities [17, 18], only maternal (but not paternal) mental illness diagnosed prior to the child’s birth was associated with membership in each of the age 5–6 year risk classes when examined simultaneously in the present study. The lack of, or smaller association with paternal mental illness is consistent with other findings in relation to externalising problems at age 5 years and emotional and behavioural development at age 3–5 years [13, 34, 35].

The main strength of this study is the large population-based sample of children and their parents, with data available during the pregnancy period﻿ [20]. The use of population-level data linkage minimises selection bias and mitigates against recall bias, while enabling the examination of rare exposures due to the large sample size. However, these findings in this study must be considered in view of several limitations. First, our index of socioeconomic disadvantage was area-based; while this is a valid approach to estimating the socioeconomic status of groups, it may be less robust as an index of individual (family) status. Second, the diagnoses of parental mental illness included only those receiving inpatient or outpatient/community-based mental health treatment, and likely underrepresents milder forms of mental illness that are treated elsewhere such as in primary care. Third, parental records were only available for children with births registered in NSW; while there is potential for this to introduce selection bias (i.e., we were unable to obtain linked parent data for children who migrated from interstate or internationally between birth and school entry), we have previously demonstrated the comparability of the sample of children with linked parental data to the entire population cohort, based on key demographic characteristics [17, 19]. Fourth, the current age of the child cohort precludes investigating the extent to which perinatal factors common to the ‘pervasive risk’ and ‘mild generalised risk’ classes may impact in later years beyond the time frame of this study [36]. For example, while there is considerable evidence supporting a link between specific perinatal factors and externalising behaviours in adolescence (e.g., low birthweight and ADHD) [37], the research examining the links between perinatal factors and internalising behaviours is mixed (e.g., preterm birth and anxiety) [38]. Fifth, some cells (i.e., grand multiparity, no antenatal visit, parental mental health and prenatal child protection contact) had relatively small sample sizes and results for these categories must therefore be interpreted cautiously. Sixth, despite male sex arising as one of the strongest risk factors for all three risk classes, this likely reflects an underrepresentation of girls as developmentally vulnerable on specific AEDC indices due to differences in the visibility of particular types of vulnerabilities; that is, externalising problems are more salient in the classroom and more commonly prevalent in boys, whereas internalising problems in children are more commonly prevalent in girls. Finally, we note that information about the ethnicity of the Australian population is not readily comparable with that recorded by other countries, since national demographic information focuses on country of birth and language background other than English, as opposed to recording identification with broad ethnic groups or race.

## Summary

The present study suggests two patterns of risk factors associated with early childhood risk classes that signify vulnerability for later mental illness and other social problems. The results indicate that certain factors measurable during pregnancy and known at the time of birth could be used to inform targeted service provision for families with these patterns of risk evident in the perinatal period, shown here to be associated with distinct profiles of early childhood developmental vulnerabilities measured at school entry. These findings highlight the potential for more effective delivery of targeted services in the first 2000 days for vulnerable children and families, to improve mental health and other social outcomes in later life.

## Data Availability

The ethical and legal conditions governing the NSW-CDS investigators' access and linkage of the Government owned datasets used in this study explicitly prohibit sharing this data with external parties.

## References

[1] World Health Organization (2003) Investing in mental health. World Health Organization.

[2] Slade T, Johnston A, Teesson M, Whiteford H, Burgess P, Pirkis J, Saw S (2009) The mental health of Australians 2. Report on the 2007 National Survey of Mental Health and *Wellbeing*. (P3-5317). Canberra

[3] Lawrence D, Hafekost J, Johnson SE, Saw S, Buckingham WJ, Sawyer MG et al (2016) Key findings from the second Australian Child and Adolescent Survey of Mental Health and Wellbeing. Aust N Z J Psychiatry 50(9):876–886. 10.1177/0004867415617836 26644606 10.1177/0004867415617836

[4] García JL, Heckman JJ, Leaf DE, Prados MJ (2020) Quantifying the life-cycle benefits of an influential early-childhood program. J Polit Econ 128(7):2502–2541. 10.1086/705718 32616965 10.1086/705718 PMC7331936

[5] Kautz T, Heckman JJ (2013) Fostering and measuring skills: interventions that improve character and cognition. NBER Working Paper Series

[6] Kim-Cohen J, Caspi A, Moffitt TE, Harrington H, Milne BJ, Poulton R (2003) Prior juvenile diagnoses in adults with mental disorder: developmental follow-back of a prospective-longitudinal cohort. Arch Gen Psychiatry 60(7):709–717. 10.1001/archpsyc.60.7.709 12860775 10.1001/archpsyc.60.7.709

[7] Productivity Commission (2020) Mental health. (Report no. 95). Canberra

[8] Department of Social Services (2018) Supporting families, communities and organisations to keep children safe: National Framework for Protecting Australia’s Children 2009–2020—Fourth Action Plan 2018–2020. (ISBN 978-1-925318-80-7). https://www.dss.gov.au/sites/default/files/documents/01_2019/dss-fourth-action-plan-v6-web-final.pdf

[9] Moore TG, Arefadib N, Deery A, West S (2017) The first thousand days: an evidence paper. Parkville, Victoria: Centre for Community Child Health, Murdoch Children’s Research Institute

[10] Dean K, Green MJ, Laurens KR, Kariuki M, Tzoumakis S, Sprague T et al (2018) The impact of parental mental illness across the full diagnostic spectrum on externalising and internalising vulnerabilities in young offspring. Psychol Med 48(13):2257–2263. 10.1017/s0033291717003786 29331151 10.1017/S0033291717003786

[11] Huhdanpää H, Morales-Muñoz I, Aronen ET, Pölkki P, Saarenpää-Heikkilä O, Kylliäinen A, Paavonen EJ (2021) Prenatal and postnatal predictive factors for children’s inattentive and hyperactive symptoms at 5 years of age: the role of early family-related factors. Child Psychiatry Hum Dev 52(5):783–799. 10.1007/s10578-020-01057-7 32951139 10.1007/s10578-020-01057-7 PMC8405488

[12] Laurens KR, Tzoumakis S, Kariuki M, Green MJ, Hamde M, Harris F et al (2017) Pervasive influence of maternal and paternal criminal offending on early childhood development: a population data linkage study. Psychol Med 47(5):889–901. 10.1017/S0033291716003007 27894371 10.1017/S0033291716003007 PMC5341495

[13] Mantymaa M, Puura K, Luoma I, Latva R, Salmelin RK, Tamminen T (2012) Predicting internalizing and externalizing problems at five years by child and parental factors in infancy and toddlerhood. Child Psychiatry Hum Dev 43(2):153–170. 10.1007/s10578-011-0255-0 21956275 10.1007/s10578-011-0255-0

[14] Robinson M, Oddy WH, Li J, Kendall GE, De Klerk NH, Silburn SR et al (2008) Pre- and postnatal influences on preschool mental health: a large-scale cohort study. J Child Psychol Psychiatry 49(10):1118–1128. 10.1111/j.1469-7610.2008.01955.x 19017026 10.1111/j.1469-7610.2008.01955.x

[15] Tuna Cak H, Gokler B (2013) Attention deficit hyperactivity disorder and associated perinatal risk factors in preterm children. Turk Pediatr Arch 48:315–322

[16] Watkeys OJ, Dean K, Laurens KR, Harris F, Carr VJ, Green MJ (2022) Familial clustering of birth risk for adverse childhood outcomes. J Perinatol 42(5):603–610. 10.1038/s41372-021-01264-7 34795406 10.1038/s41372-021-01264-7

[17] Green MJ, Tzoumakis S, Laurens KR, Dean K, Kariuki M, Harris F et al (2019) Early developmental risk for subsequent childhood mental disorders in an Australian population cohort. Aust N Z J Psychiatry 53(4):304–315. 10.1177/0004867418814943 30501395 10.1177/0004867418814943

[18] Dean K, Whitten T, Tzoumakis S, Laurens KR, Harris F, Carr VJ, Green MJ (2021) Incidence of early police contact among children with emerging mental health problems in Australia. JAMA Netw Open 4(6):e2112057–e2112057. 10.1001/jamanetworkopen.2021.12057 34156455 10.1001/jamanetworkopen.2021.12057 PMC8220465

[19] Green MJ, Tzoumakis S, Laurens KR, Dean K, Kariuki M, Harris F et al (2018) Latent profiles of early developmental vulnerabilities in a New South Wales child population at age 5 years. Aust N Z J Psychiatry 52(6):530–541. 10.1177/0004867417740208 29108437 10.1177/0004867417740208

[20] Green MJ, Harris F, Laurens KR, Kariuki M, Tzoumakis S, Dean K et al (2018) Cohort profile: the New South Wales Child Development Study (NSW-CDS)—wave 2 (child age 13 years). Int J Epidemiol 47(5):1396–1397k. 10.1093/ije/dyy115 30016428 10.1093/ije/dyy115

[21] Australian Bureau of Statistics (2022) Cultural diversity of Australia. https://www.abs.gov.au/articles/cultural-diversity-australia

[22] Australian Bureau of Statistics (2006) Socio-economic indexes for areas (SEIFA). ABS Cat. No. 2033.0.55.001. https://www.abs.gov.au/ausstats/abs@.nsf/mf/2033.0.55.001

[23] Alwan NA, Roderick PJ, Macklon NS (2016) Is timing of the first antenatal visit associated with adverse birth outcomes? Analysis from a population-based birth cohort. The Lancet 388:S18. 10.1016/S0140-6736(16)32254-1

[24] Dobbins TA, Sullivan EA, Roberts CL, Simpson JM (2012) Australian national birthweight percentiles by sex and gestational age, 1998–2007. Med J Aust 197(5):291–294. 10.5694/mja11.11331 22938128 10.5694/mja11.11331

[25] Brinkman SA, Silburn S, Lawrence D, Goldfeld S, Sayers M, Oberklaid F (2007) Investigating the validity of the Australian Early Development Index. Early Educ Dev 18(3):427–451. 10.1080/10409280701610812

[26] Janus M, Brinkman S, Duku E (2011) Validity and psychometric properties of the early development instrument in Canada, Australia, United States, and Jamaica. Soc Indicators Res 103(2):283–297

[27] Brinkman SA, Gregory TA, Goldfeld S, Lynch JW, Hardy M (2014) Data resource profile: the Australian Early Development Index (AEDI). Int J Epidemiol 43(4):1089–1096. 10.1093/ije/dyu085 24771275 10.1093/ije/dyu085 PMC4258780

[28] IBM (2019) IBM SPSS statistics for windows (version 26.0). IBM Corp, Armonk

[29] Taplin S (2017) Prenatal reporting to child protection: characteristics and service responses in one Australian jurisdiction. Child Abuse Negl 65:68–76. 10.1016/j.chiabu.2017.01.007 28113086 10.1016/j.chiabu.2017.01.007

[30] NSW Department of Health (2009) NSW Health/Families NSW Supporting Families Early Package—maternal and child health primary health care policy. Sydney, Australia. http://www.sfe.nswiop.nsw.edu.au/file.php/1/NSWHEALTH_SFEmaternal_ChildHealth.pdf

[31] Goldfeld S, O’Connor E, O’Connor M, Sayers M, Moore T, Kvalsvig A, Brinkman S (2016) The role of preschool in promoting children’s healthy development: evidence from an Australian population cohort. Early Childhood Research Quarterly 35:40–48. 10.1016/j.ecresq.2015.11.001

[32] Heckman JJ (2011) The economics of inequality: the value of early childhood education. Am Educ 35(1):31–47

[33] Taylor CL, Christensen D, Jose K, Zubrick SR (2022) Universal child health and early education service use from birth through Kindergarten and developmental vulnerability in the Preparatory Year (age 5 years) in Tasmania, Australia. Aust J Soc Issues 57:289–313. 10.1002/ajs4.186 10.23889/ijpds.v6i3.1681 PMC8780991 35136844

[34] Kvalevaag AL, Ramchandani PG, Hove O, Eberhard-Gran M, Assmus J, Havik OE et al (2015) Parents’ prenatal mental health and emotional, behavioral and social development in their children. Child Psychiatry Hum Dev 46(6):874–883. 10.1007/s10578-014-0527-6 25504529 10.1007/s10578-014-0527-6

[35] Mensah FK, Kiernan KE (2010) Parents’ mental health and children’s cognitive and social development. Soc Psychiatry Psychiatr Epidemiol 45(11):1023–1035. 10.1007/s00127-009-0137-y 19823757 10.1007/s00127-009-0137-y

[36] Schoon I, Melis G (2019) Intergenerational transmission of family adversity: examining constellations of risk factors. PLoS ONE 14(4):e0214801–e0214801. 10.1371/journal.pone.0214801 31017914 10.1371/journal.pone.0214801 PMC6481806

[37] Latimer K, Wilson P, Kemp J, Thompson L, Sim F, Gillberg C et al (2012) Disruptive behaviour disorders: a systematic review of environmental antenatal and early years risk factors. Child 38(5):611–628. 10.1111/j.1365-2214.2012.01366.x 10.1111/j.1365-2214.2012.01366.x 22372737

[38] Ståhlberg T, Khanal P, Chudal R, Luntamo T, Kronström K, Sourander A (2020) Prenatal and perinatal risk factors for anxiety disorders among children and adolescents: a systematic review. J Affect Disord 277:85–93. 10.1016/j.jad.2020.08.004 32799108 10.1016/j.jad.2020.08.004

